# Identification of drug candidate for osteoporosis by computational bioinformatics analysis of gene expression profile

**DOI:** 10.1186/2047-783X-18-5

**Published:** 2013-03-01

**Authors:** Guiyong Yu, Litao Wang, Yazhou Li, Zhihong Ma, Yu Li

**Affiliations:** 1 Department of Orthopedic, The people's Hospital of Hengshui, No.180 Renmin Street, 053000, Hebei Province, Hengshui, China; 2 Department of Orthopedic, The Third people's Hospital of Hebei Province, Hebei Province, China; 3 Clinical Laboratory, The Second people's Hospital of Tangshan, Hebei Province, China

**Keywords:** Osteoporosis, Differentially expressed genes, Dysfunctional pathway, Small molecule

## Abstract

**Background:**

Osteoporosis is a condition of bones that leads to an increased susceptibility to fracture and consequent painful morbidity. It has become a major issue of life quality worldwide. However, until now, the molecular mechanism of this disease is far from being clear.

**Methods:**

In this study, we obtained the gene expression profile of osteoporosis and controls from Gene Expression Omnibus and identified differentially expressed genes (DEGs) using classical t-test method. Then, functional enrichment analyses were performed to identify the dysregulated Gene Ontology categories and dysfunctional pathways in osteoporosis patients compared to controls. Besides, the connectivity map was used to identify compounds that induced inverse gene changes to osteoporosis.

**Results:**

A total of 5581 DEGs were identified. We found these DEGs were enriched in 9 pathways by pathway enrichment analysis, including focal adhesion and MAPK signaling pathway. Besides, sanguinarine was identified as a potential therapeutic drug candidate capable of targeting osteoporosis.

**Conclusion:**

Although candidate agents identified by our approach may be premature for clinical trials, it is clearly a direction that warrants additional consideration.

## Background

Osteoporosis is a condition of bones that leads to an increased susceptibility to fracture and consequent painful morbidity [1,2]. The prevalence of osteoporosis increases with age due to an imbalance between bone resorption and bone formation during the bone remodeling cycle. Osteoporosis affects up to 30% of women and 12% of men at some point in life and it is a major quality of life issue worldwide [3].

The well accepted pathophysiological mechanisms for osteoporosis include early apoptosis of osteoblasts [4] and osteocytes [5,6], prolongation of the life span of osteoclasts [7] and the imbalance between osteoblastogenesis and adipogenesis of bone marrow mesenchymal stem cells [8]. Many factors influence the risk of osteoporosis, including predominantly peak bone density along with other factors such as genetic factors, body weight, diet, physical activity, medication use, and coexisting disease [3,9,10]. Besides, lack of estrogen, deficiency of calcium and vitamin D are also important common causes of osteoporosis [11-14]. Various molecular signals were identified to regulate the activation of osteoclasts. Osteoprotegerin (OPG) binds activator for nuclear factor κB ligand (RANKL), and hence suppresses its ability to increase bone resorption [15]. The role of Wnt signaling pathway is recognized but less well understood. Local production of eicosanoids and interleukins is thought to participate in the regulation of bone turnover, and excess or reduced production of these mediators may underlie the development of osteoporosis [16]. However, until now, the molecular mechanism of this disease is far from being clear.

In the present study, we aim to explore the molecular mechanism of osteoporosis using a computational bioinformatics analysis of gene expression, and to identify small molecules for the treatment of osteoporosis. Candidate agents identified by our approach may provide the ground work for a new therapy approach for osteoporosis. However, further evaluations for their potential use are needed.

## Methods

### Affymetrix microarray data

The gene expression profile of GSE 35956 [17] was downloaded from a public functional genomics data repository Gene Expression Omnibus (GEO; http://www.ncbi.nlm.nih.gov/geo/), which is based on the Affymetrix GPL570 platform data (Affymetrix Human Genome U133 Plus 2.0 Array). Only 10 genechips were available for further analysis, including 5 genechips from human mesenchymal stem cells (MSCs) of osteoporosis patients and 5 genechips from human MSCs of non-osteoporotic controls. The Human MSCs of elderly patients suffering from osteoporosis were isolated from femoral heads after low-energy fracture of the femoral neck. Control cells were obtained from bone marrow of femoral heads of middle-aged, non-osteoporotic donors after total hip arthroplasty.

### Pathway data

KEGG (Kyoto Encyclopedia of Genes and Genomes) is a collection of online databases dealing with genomes, enzymatic pathways, and biological chemicals [18]. The PATHWAY database records networks of molecular interactions in the cells, and variants of them specific to particular organisms (http://www.genome.jp/kegg/).

### Small molecules data

The connectivity map (CMap) can be used to find connections among small molecules sharing a mechanism of action, chemicals and physiological processes, and diseases and drugs [19]. It is the first installment of a reference collection of gene-expression profiles from cultured human cells treated with bioactive small molecules, together with pattern-matching software to mine these data. The CMap dataset comprises genomic profiling data from 6100 treatment-control pairs (instances) involving 1309 bioactive molecules (perturbagens). We downloaded all the profile data for further analysis.

### Differentially expressed genes (DEG) analysis

For GSE 35956, we used R package (v.2.13.0) to analyze the gene expression. The CEL source files from all conditions were processed into expression estimates and performed background correction and quartile data normalization using RMA (Robust Multi-array Average) algorithm [20]. Probe sets were mapped to national center for biotechnology information (NCBI) entrez genes using DAVID [21]. If there are multiple probe sets that correspond to the same gene, the expression values of those probe sets are averaged. We used the classical t-test to identify differentially expressed genes and defined p-value < 0.05 to be statistically significant.

### Go ontology analysis

Gene Ontology (GO) analysis has become a commonly used approach for functional studies of large-scale genomic or transcriptomic data [22]. To better understand the functional relevance of the identified DEGs, we performed GO enrichment analysis using goProfiles [23] and searched for over-representation in GO categories in three categories, namely biological process, molecular function and cellular component.

### Pathway enrichment analysis

DAVID (The Database for Annotation, Visualization and Integrated Discovery) consists of an integrated biological knowledgebase and analytic tools aimed at systematically extracting biological meaning from large gene/protein lists [21]. We used the DAVID to identify over-represented KEGG categories in pathways based on the hypergeometric distribution with the count larger than 2 and the FDR less than 0.01.

### Small molecule identification

We first divided the DEGs into two groups: up-regulated group and down-regulated group. Then, we selected the top 500 significant DEGs in each group and performed gene set enrichment analysis compared to the gene profile of a treatment-control pair (instance) in CMap database. The output consisted of a group of chemical perturbagens with an enrichment score ranging from +1 and −1. The score represented the correlation between the query signature profile (osteoporosis) and the gene profile of a treatment-control pair. A high positive connectivity score indicated that the corresponding perturbagen induced the expression of the query signature (osteoporosis), whereas a high negative enrichment score indicated reversal of expression of the query signature (osteoporosis) by the perturbagen. A zero or “null” enrichment score indicated that no effect upon expression of the query signature was recorded.

## Results

### Analysis of DEGs in osteoporosis

Publicly available microarray dataset of human MSCs from osteoporosis and control were obtained from GEO database. We used the classical t-test method to calculate the p-values of all genes and defined p-value < 0.05 to be statistically significant. Expressions of 5581 genes were identified differed across osteoporosis and control group.

### Functional annotation of the DEGs

**Figure 1:**
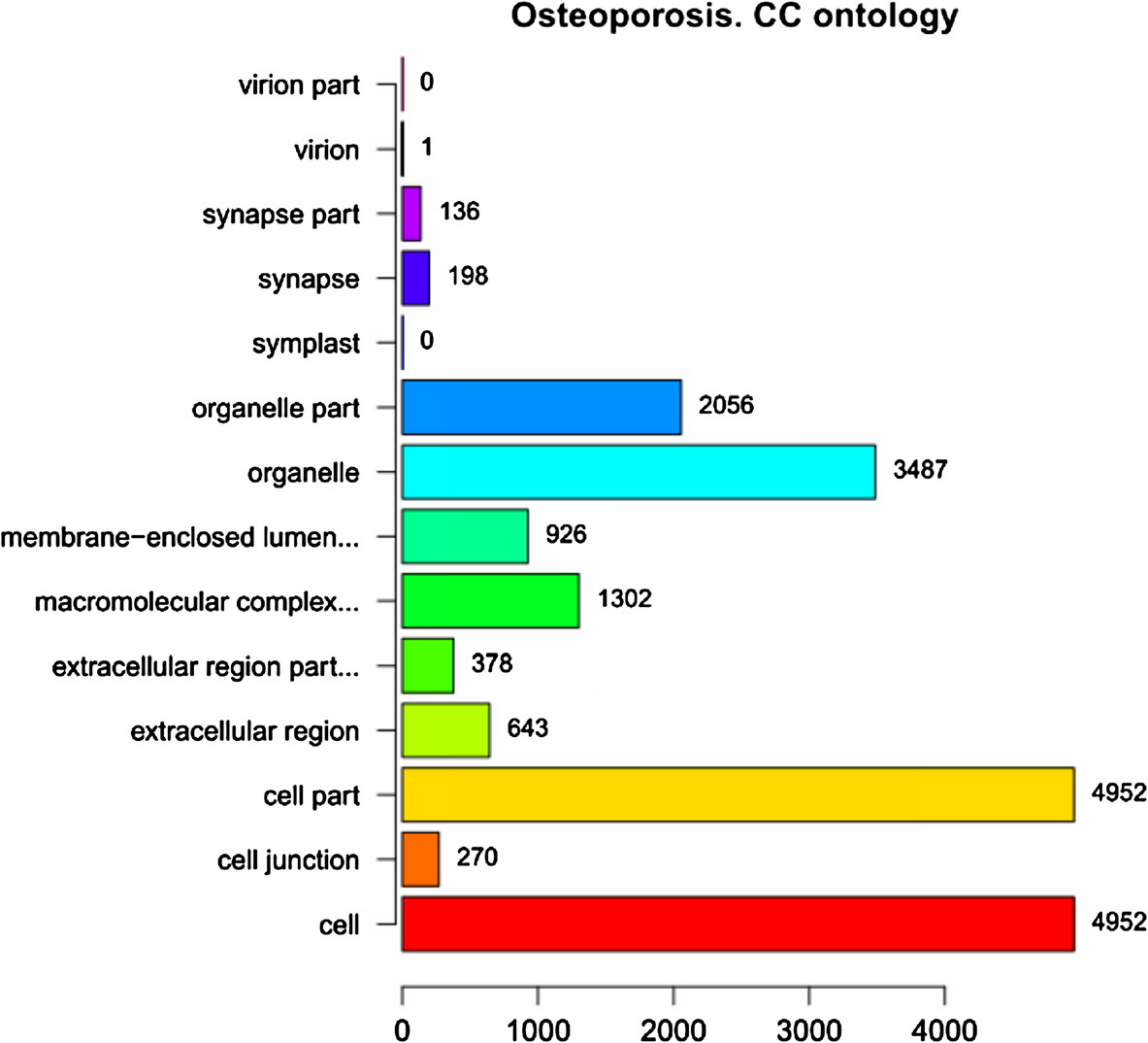
**GO Enrichment of DEGs in cellular component ontology.** The ordinate indicates the enriched GO categories in cellular component ontology and the abscissa indicates the number of DEGs in each category.

**Figure 2:**
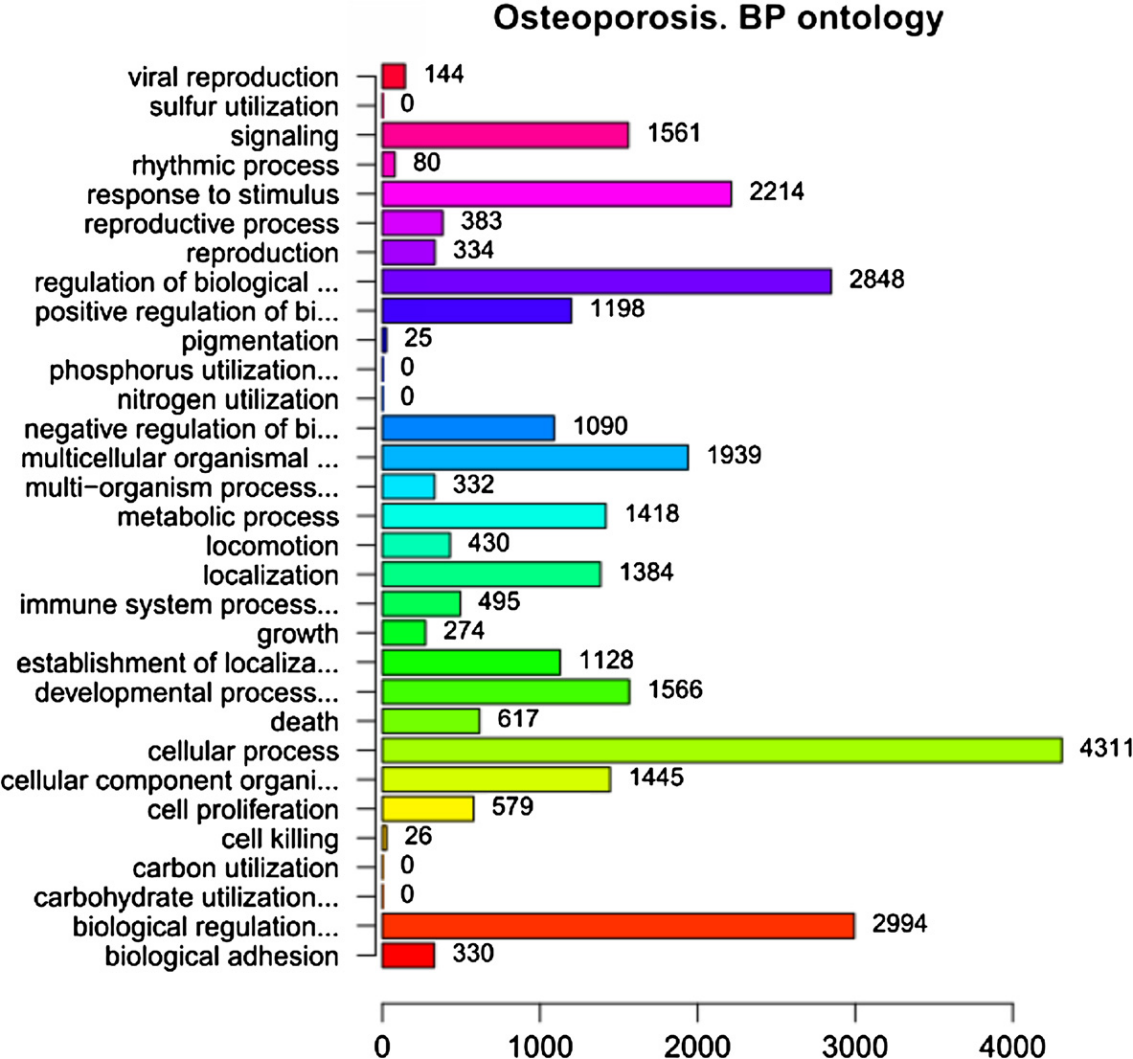
**GO Enrichment of DEGs in biological process ontology.** The ordinate indicates the enriched GO categories in biological process ontology and the abscissa indicates the number of DEGs in each category.

**Figure 3:**
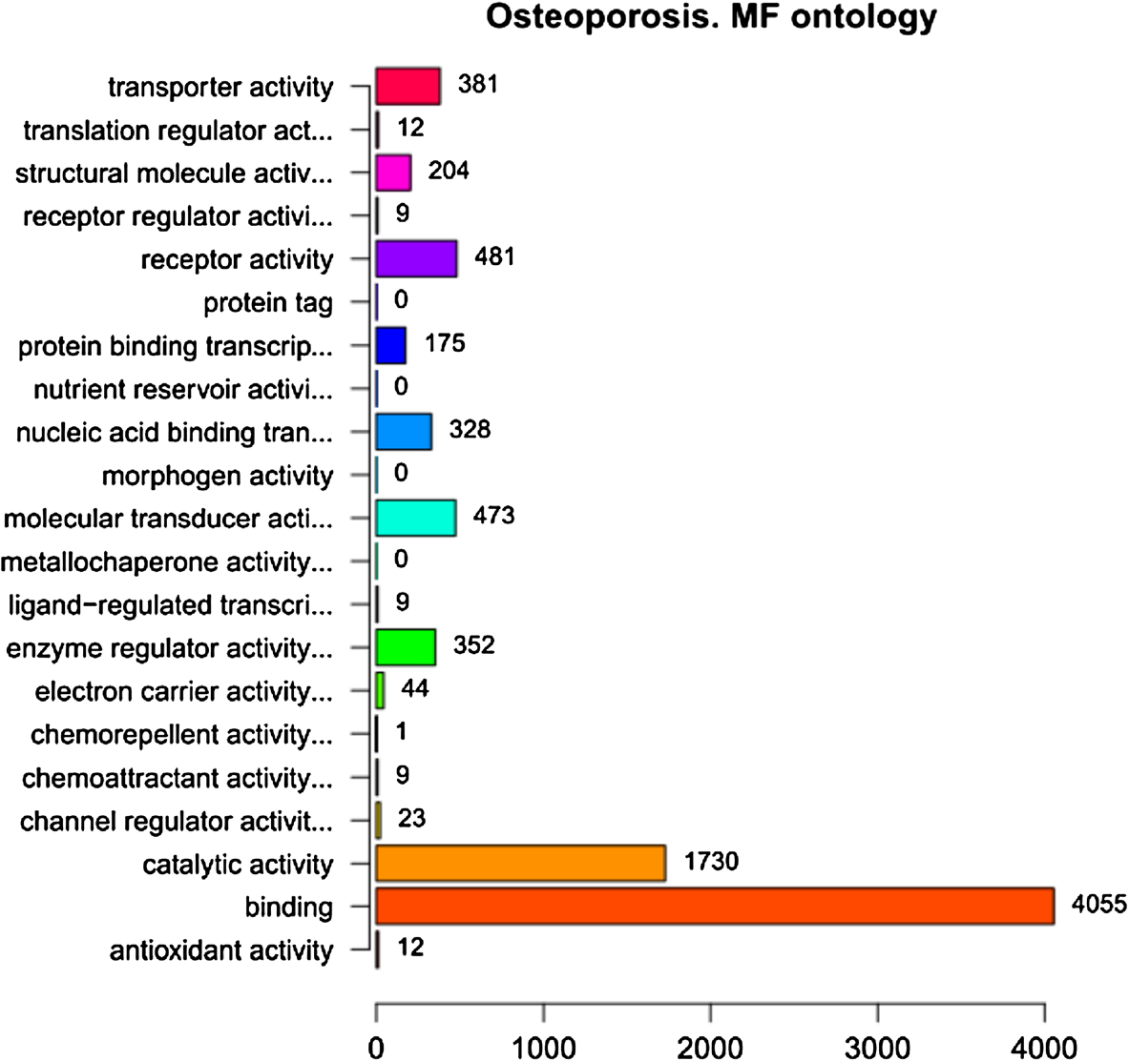
**GO Enrichment of DEGs in molecular function ontology.** The ordinate indicates the enriched GO categories in molecular function ontology and the abscissa indicates the number of DEGs in each category.

In order to facilitate the functional annotation and analysis of large lists of genes in our result, we identified over-represented GO categories in three ontologies (Figure 1, Figure 2 and Figure 3). In the ontology of cellular component, GO categories of cell and cell part tied for first place in the enrichment (4982 genes). Besides, a total of 3487 genes were enriched in the GO category of organelle. In the ontology of biological process, the most significant GO category was cellular process, and a total of 4311 genes enriched in this category. In the ontology of molecular function, the GO category of binding included 4055 genes which was the most significant category in this ontology. The second significant GO category is catalytic activity (1730 genes).

### Identification of dysfunctional pathways in osteoporosis

We performed pathway enrichment analysis using the online biological classification tool DAVID. A total of 9 dysfunctional pathways with p-value less than 0.01 were enriched (Table 1). The most significant dysfunctional pathway was focal adhesion with p-value = 5.04E-04. The other significant pathways included MAPK signaling pathway (p-value = 0.001953), allograft rejection (p-value = 0.003975) and DNA replication (p-value= 0.003975).

**Table 1:** The enriched KEGG pathways

**KEGG-ID**	**Description**	**P-Value**
hsa04510	Focal adhesion	5.04E-04
hsa04010	MAPK signaling pathway	0.001953
hsa05330	Allograft rejection	0.003975
hsa03030	DNA replication	0.003975
hsa05211	Renal cell carcinoma	0.004548
hsa04912	GnRH signaling pathway	0.005447
hsa04720	Long-term potentiation	0.005452
hsa04110	Cell cycle	0.007658
hsa04120	Ubiquitin mediated proteolysis	0.008467

### Identification of candidate small molecules

In order to identify candidate small molecules capable to reverse the gene changes of osteoporosis, we performed computational bioinformatics analysis of the derived gene signature using the Connectivity Map. The perturbagens from the CMap were analyzed according to their permutated results, p-values, and enrichment scores. A search against 6100 treatment-control pairs (instances) representing 1309 bioactive small molecules identified large amount small molecules which exhibited positive or negative correlation to the query signature. The top 20 significant small molecules were listed in Table 2. In Table 2, the small molecule of sanguinarine (enrichment score = −0.968) was associated with highly significant negative scores and the small molecule of isoflupredone was associated with highly significant positive score (enrichment score = 0.95).

**Table 2:** Small molecules capable to reverse osteoporosis

**Perturbagen**	**Enrichment score**	**p-value**
viomycin	0.915	0.00004
isoflupredone	0.95	0.00012
adiphenine	0.845	0.00018
medrysone	−0.754	0.00044
luteolin	−0.874	0.00054
thiamphenicol	0.806	0.00062
sulconazole	−0.86	0.00068
apigenin	−0.857	0.00074
cinchonine	0.849	0.00074
thioguanosine	−0.852	0.00092
genistein	0.459	0.00092
roxithromycin	−0.843	0.00105
GW-8510	−0.842	0.00107
nadolol	0.827	0.00135
phthalylsulfathiazole	−0.751	0.00174
rimexolone	−0.825	0.00177
trazodone	−0.899	0.00194
sanguinarine	−0.968	0.00221
camptothecin	−0.895	0.00224
podophyllotoxin	0.807	0.00257

## Discussion

Gene expression profiling in disease reveals the underlying gene activity changes contributing to the disease and enables targets for therapeutic intervention to be identified. In this study, we investigated gene expression profile in human MSCs from patients of osteoporosis and controls, and then identified biologically active small molecules capable to reverse gene changes of osteoporosis using computational bioinformatics methods. Results show that a total of 5581 genes were differentially expressed between osteoporosis and controls. In addition, we identified large amount of small molecules which can provide new ideas for the therapeutic studies in osteoporosis.

Up to 5581 genes were identified differentially expressed between osteoporosis and control in our approach. These DEGs may play critical roles in the initiation of osteoporosis, and investigation of them may shed new lights on understanding of the molecular mechanism of osteoporosis. Pathway enrichment analysis of these DEGs indicated a total of 9 pathways were dysregulated in the development of osteoporosis, including focal adhesion and MAPK signaling pathway.

Focal adhesions, which are specialized sites of attachment between cells and the extracellular matrix, play a role in cell motility, cell proliferation, signal transduction and have been proposed to function as mechanosensors [24-26]. Osteoporosis is a result of an imbalance of bone formation and resorption. In osteoporosis, the regenerative capacity of bone is compromised, which may involve altered osteoblast activity. This could be attributed to an inappropriate synthesis and assembly of an extracellular matrix (ECM), altered cell adhesion to the ECM, or be due to inappropriate downstream activation of adhesion-mediated signaling cascades through proteins such as focal adhesion kinase (FAK). Perinpanayagam *et al*. suggested that early adhesion-mediated events, such as cell adhesion, attachment, and FAK signaling may be altered in osteoporotic osteoblast cells [27]. In our result, focal adhesion was the most significant dysfunctional pathways in the initiation of osteoporosis.

MAPK signaling pathways transduces a large variety of external signals, leading to a wide range of cellular responses, including growth, differentiation, inflammation and apoptosis [28]. Several studies have suggested that MAPK signaling pathways contribute greatly to osteoblast differentiation and bone formation via TGF-β and bone morphogenic protein (BMP) signaling pathways. Lee *et al*. demonstrated that MAPK pathways converge at the Runx2 gene to control mesenchymal precursor cell differentiation following TGF-β induction [29]. Recent study revealed that TGF-β signaling promotes osteoprogenitor proliferation, early differentiation, and commitment to the osteoblastic lineage through the selective MAPKs pathways [30]. In addition, MAPK dependent phosphorylation, TGF-β/BMP signaling, and Runx2 subnuclear targeting converge to induce the osteogenic phenotype [31].

The identification of a group of small molecules with potential therapeutic efficacy for osteoporosis is an important observation of our work. Data in Table 2 shows that sanguinarine (enrichment score = −0.968) was associated with highly significant negative score, suggesting that this small molecule is capable of targeting osteoporosis. Sanguinarine, a component of sanguinaria extract, has been shown to display antitumor and anti-inflammatory properties in animals [32,33] and to inhibit neutrophil function [34]. Madan *et al*. demonstrate that sanguinarine is a potent suppressor of NF-kB activation that blocks the phosphorylation and degradation of IkBα [35]. Recently, it was discovered that the RANK/RANKL/OPG system is an important signal transduction pathway that regulates osteoclast formation [36]. Targeting of this pathway is a novel therapeutic approach in the management of osteoporosis. Therefore, sanguinarine may provide promising targets for the future development of novel treatments of osteoporosis. However, further evaluation for their potential use in the treatment of osteoporosis is still needed.

## Conclusion

In conclusion, we have identified a total of 9 dysfunctional pathways in the development of osteoporosis. Among them, focal adhesion and MAPK signaling pathway were the most significant ones. Besides, we have identified that sanguinarine may be a therapeutic drug candidate capable of targeting osteoporosis. Although it may be premature to suggest that this drug might be ready for clinical trials, it is clearly a direction that warrants additional consideration.

## Abbreviations

DEGs: Differentially expressed genes;MSCs: Mesenchymal stem cells;KEGG: Kyoto Encyclopedia of Genes and Genomes;CMap: Connectivity map;GO: Gene Ontology;DAVID: The Database for Annotation, Visualization and Integrated Discovery;ECM: Extracellular matrix;FAK: Focal adhesion kinase;BMP: Bone morphogenic protein

## Competing interests

The authors declare that they have no competing interests.

## Authors’ contributions

Design and acquire the data: GY, LW. Analyze and interpret the data: GY, LW, YL, ZM. Draft and revise the manuscript: GY, YL. All authors read and approved the final manuscript.
